# Evaluation of Transducer Elements Based on Different Material Configurations for Aptamer-Based Electrochemical Biosensors

**DOI:** 10.3390/bios14070341

**Published:** 2024-07-13

**Authors:** Ivan Lopez Carrasco, Gianaurelio Cuniberti, Jörg Opitz, Natalia Beshchasna

**Affiliations:** 1 Fraunhofer Institute for Ceramic Technologies and Systems IKTS, Maria-Reiche-Strasse 2, 01109 Dresden, Germany; ivan.lopez.carrasco@ikts.fraunhofer.com (I.L.C.); joerg.opitz@ikts.fraunhofer.de (J.O.); 2 Faculty of Mechanical Science and Engineering, Institute of Materials Science and Max Bergmann Center of Biomaterials, Technische Universität Dresden, 01062 Dresden, Germany; office.nano@tu-dresden.de

**Keywords:** electrochemical biosensor, gold electrodes, aptamer, cardiac troponin, EIS, CV

## Abstract

The selection of an appropriate transducer is a key element in biosensor development. Currently, a wide variety of substrates and working electrode materials utilizing different fabrication techniques are used in the field of biosensors. In the frame of this study, the following three specific material configurations with gold-finish layers were investigated regarding their efficacy to be used as electrochemical (EC) biosensors: (I) a silicone-based sensor substrate with a layer configuration of 50 nm SiO/50 nm SiN/100 nm Au/30–50 nm WTi/140 nm SiO/bulk Si); (II) polyethylene naphthalate (PEN) with a gold inkjet-printed layer; and (III) polyethylene terephthalate (PET) with a screen-printed gold layer. Electrodes were characterized using electrochemical impedance spectroscopy (EIS) and cyclic voltammetry (CV) to evaluate their performance as electrochemical transducers in an aptamer-based biosensor for the detection of cardiac troponin I using the redox molecule hexacyanoferrade/hexacyaniferrade (K3[Fe (CN)6]/K4[Fe (CN)6]. Baseline signals were obtained from clean electrodes after a specific cleaning procedure and after functionalization with the thiolate cardiac troponin I aptamers “Tro4” and “Tro6”. With the goal of improving the PEN-based and PET-based performance, sintered PEN-based samples and PET-based samples with a carbon or silver layer under the gold were studied. The effect of a high number of immobilized aptamers will be tested in further work using the PEN-based sample. In this study, the charge-transfer resistance (Rct), anodic peak height (I_pa_), cathodic peak height (I_pc_) and peak separation (∆E) were determined. The PEN-based electrodes demonstrated better biosensor properties such as lower initial Rct values, a greater change in Rct after the immobilization of the Tro4 aptamer on its surface, higher I_pc_ and I_pa_ values and lower ∆E, which correlated with a higher number of immobilized aptamers compared with the other two types of samples functionalized using the same procedure.

## 1. Introduction

Biosensors are analytical devices that combine two main elements, a biorecognition element and a transducer. The device recognizes certain biological phenomena and translates them into measurable signals. Biosensors have a wide range of applications, including healthcare diagnostics, drug discovery, biomedicine, food processing and safety and environmental monitoring. These areas are made possible by the selection of appropriate biorecognition elements and transducers. Since the development of the first biosensors, scientists have worked closely with new types of bioreceptors, transducers, immobilization protocols and transducer manufacturing technologies to produce biosensors that are reliable and inexpensive, with low detection limits and high specificity [1,2,3,4].

There are several classifications and subclassifications that can be used for different biosensors. The classification can be based on the type of transducer or the biorecognition element used. If the detection mechanism is considered, we have a biocatalytic group, including enzymes; a bioaffinity group, including antibodies and nucleic acids; and a microbe-based group containing microorganisms. If the transducer type is considered, the main classifications of biosensors include mass sensitive, optical, electrochemical and thermal [5].

Currently, there are several commercially available biosensors; examples include enzyme-based and tissue-based as well as immunosensors, DNA biosensors, thermal and piezoelectric biosensors [6]. In general, the field of biosensors continues to grow as studies mention that the global biosensor market size was valued at USD 26.8 billion in 2022 and is expected to grow at a compound annual growth rate (CAGR) of 8.0% from 2023 to 2030 [7]. Some of the more specific drivers of this growth are the prevalence of chronic diseases such as cancer, HIV, antimicrobial resistance (AMR) and cardiovascular problems. Here, electrochemical biosensors have an opportunity because they can be precise, fast and non-invasive, with other advantages such as low production costs, robustness, miniaturization capabilities and ease of use for diagnostic and monitoring purposes [8,9]. The market for electrochemical biosensors is expected to have a global CAGR of 6.65% and reach 26.8 billion USD by 2030. The European region has the second highest market share [10].

**Figure 1:**
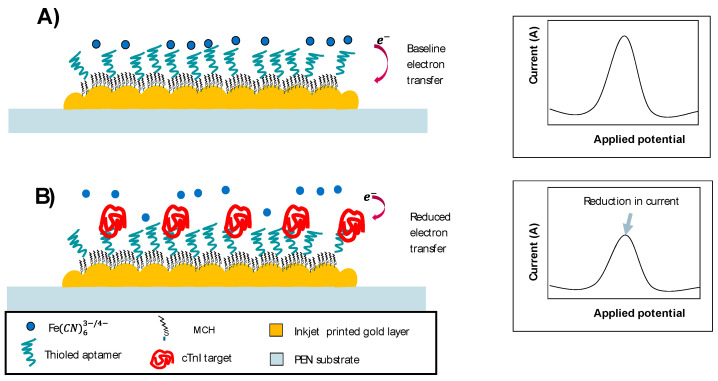
General scheme for the label-free aptamer-based sensors used to detect cardiac troponin I. (**A**) Graphical representation of aptamer-based sensor electron transfer process without target and recoding of signal without target binding to the aptamer-modified surface using [Fe (CN)6]^3−/4−^ redox probe freely in a solution (baseline signal). (**B**) Graphical representation of aptamer-based sensor electron transfer process with target and recoding of signal with target bound to the aptamer-modified surface using [Fe (CN)6]^3−/4−^ redox probe freely in a solution (reduced signal).

The electrochemical category of biosensors uses an electrochemical transducer that converts the transducer signal to an electronic signal and amplifies it. A computer then converts the signal into a physical parameter that can be interpreted and presented to the user. The direct monitoring of the analyte or the biological activity associated with the analyte are the two measurement approaches. Based on the operating principle of the electrochemical biosensor, the electrochemical transducer or electrode are the key components used to generate detectable signals that are the product of the interaction between the target and immobilized molecules. We aimed to investigate the electrochemical properties of different gold transducers fabricated using different technologies and using different materials as substrates. The selected materials included silicon, which has been used in electronics for years due to its mechanical strength and resilience against harsh environments. Other advantages include uniform structural attributes, the number of processing approaches available and surfaces modifications with other materials when use as substrates as well as access to miniaturization, a lightweight nature, biocompatibility and control at a microscale level [11,12]. The other materials used as substrates were PET and PEN, which have the potential to upscale production due to their mechanically flexible properties with no measurable changes that are high in demand. These materials present a low-cost alternative, with low-temperature manufacturing, a light weight and the easy integration of a gold layer using printing technologies [13]. Such properties open the doors to the possibility of using these materials in wearable applications [14,15]. The selected electrodes enabled us to choose the most suitable option as a starting electrode for the development of an aptamer-based electrochemical sensor to detect cardiac biomarkers. The cardiac biomarker chosen was cardiac troponin I (cTnI); an increase in its normal concentration indicates cardiac muscle damage [16]. The selected electrode was used as a label-free approach for the electrochemical detection of cTnI in biological samples; for sensors with a strong and reliable reference signal, further modifications of the sensor surface with a target solution can be identified using changes in the reference signal. The general approach was that we generated baseline signals with techniques such as DPV or SWV using a blank aptamer-modified sensor and [Fe (CN)6]^3−/4−^ freely in the solution (see Figure 1A). Then, the target was incubated on the sensor surface. After the incubation, a new electrochemical characterization was performed to determine the change in signal (see Figure 1B). The same approach was applied to the other tests.

## 2. Materials and Methods

### 2.1. Materials

Potassium hydroxide (KOH), hydrogen peroxide (30% H_2_O_2_), phosphate-buffered saline (PBS), magnesium chloride (MgCl_2_), potassium ferricyanide K3[Fe (CN)6] and potassium ferrocyanide K4[Fe (CN)6] were purchased from Carl Roth (Karlsruhe, Germany). Aptamers Tro4 and Tro6 were purchased from Eurofins Genomic (https://www.eurofins.de/, accessed on 3 July 2024, Ebersberg, Germany) and 6-mercapto-1-hexanol (MCH) was purchased from Sigma-Aldrich. All solutions were prepared in ultrapure water (18.2 MΩ.cm at 25 °C) produced using a Direct-UV Water Purification System purchased from Merck (Darmstadt, Germany).

### 2.2. Working Electrodes

Three types of electrodes manufactured using different technologies were tested as working electrodes. All electrode substrates had dimensions of 34 mm × 10 mm. The gold sensing layer had a geometrical area of 0.28 cm^2^ in a circular shape. The first type of electrode used PEN as a substrate and had an inkjet-printed gold layer 125 µm in thickness and a resistance of 20 to 30 ohms. Three batches were tested. Batch one had a non-sintered gold layer (the printer had a dpi of 846), batch two had a sintering process at 220 °C for 30 min and the dpi was 846 and batch three had a sintering process at 220 °C for 30 min and the dpi was 1016. The PEN-based electrodes were fabricated at the Fraunhofer Institute for Ceramic Technologies and Systems IKTS (Dresden, Germany). The second type used PET as the substrate and used paste for the gold layer with a gold thickness of 1 µm. Three batches were considered. The first batch comprised the PET-based electrode with gold paste only. The second batch comprised PET-based electrodes with silver paste as the first layer and a gold layer dropcasted onto the silver layer. The third batch comprised PET-based electrodes with carbon paste as the first layer and a gold layer dropcasted onto the carbon layer. The PET-based electrodes were fabricated at Innome GmBH (Dresden, Germany).

The last type of electrodes comprised silicon-based electrodes with a layer configuration of 50 nm SiO/50 nm SiN/100 nm Au/30–50 nm WTi/140 nm SiO/bulk that were fabricated by TU Dresden (https://tu-dresden.de/, Dresden, Germany).

A POSTAT204 potentiostat from Metrohm Autolab (Stuttgart, Germany) was used for the electrochemical characterization and electrochemical cleaning, with the following three-electrode configuration: the working electrode (WE) comprised the gold electrode samples, a platinum rod was the counter electrode (CE) and the Ag/AgCl electrode was the reference electrode. The electrochemical cell used in this study was designed and fabricated at the Fraunhofer Institute for Ceramic Technologies and Systems IKTS (Dresden, Germany).

### 2.3. Methods

#### 2.3.1. Cleaning Procedure

The electrodes were immersed in a beaker of ethanol and sonicated for 2 min, then rinsed with distilled water (DW) and dried with nitrogen. Once dry, the electrodes were placed in a UV/ozone cleaner for 30 min to remove organic compounds (e.g., water, carbon dioxide and nitrogen). After the UV/ozone cleaner, the electrodes were chemically cleaned in a beaker using a 3:1 solution of 0.05 M KOH and 0.05 M H_2_O_2_ for 10 min, shaking the beaker gently during the 10 min. The electrodes were then removed, rinsed with DW and dried with nitrogen. The final step was an electrochemical cleaning procedure consisting of a single linear potential sweep from −200 mV to 1200 mV [vs. Ag/AgCl (sat. 4 M KCl)] in 50 mM KOH at a sweep rate of 50 mV/s. They were thoroughly rinsed with ultrapure water (double-distilled water; Milli-Q water) and dried with N_2_. After this step, they were ready for the electrochemical characterization.

#### 2.3.2. Electrochemical Characterization

Cyclic voltammetry and electrochemical impedance spectroscopy were the two techniques used in this study for the characterization of the bare and functionalized samples. All CV and EIS experiments were performed using a standard PBS buffer The EIS measurements were obtained using at open circuit potential (OCP) and the frequency range from 100 kHz to 0.1 Hz, E_ac_ set at 0.01 Vrms; in total 61 frequencies were measured. Cyclic voltammetry (CV) measurements were obtained by sweeping a potential range from −0.1 V to +0.5 V at 0.02 V/s with a step of 0.003 V.

#### 2.3.3. Functionalization Procedure

The functionalization of the gold surfaces with Tro4 and Tro6 was carried out using a thiol anchor via a dropcasting approach. First, the stock solution of the aptamer (100 uM) was diluted to the final working concentration of 25 µM in PBS (1 mM MgCl_2_). A 10 µL drop of the aptamer solution was deposited onto a dried sample. The sample was stored in a sealed container at 4 °C for 20 h. After this time, the sample was rinsed with ultrapure water (MilliQ-water) to remove non-immobilized aptamers. A drop of 20 µL of 1 mM MCH was then applied to the aptamer-modified surface to block the exposed areas and prevent unwanted interactions with the surface. The passivation with MCH took 2 h at room temperature. The remaining MCH solution was removed after rinsing the sample with MilliQ-water. After this final step, the sample was ready to be characterized.

## 3. Results

The characterization and comparison were carried out at different stages in the development of the aptamer-based sensor. The stages considered included bare electrodes after the cleaning stage and functionalized samples using the aptamers Tro4 and Tro6. The electrodes used in this study included PEN-based electrodes, silicon-based electrodes and PET-based electrodes. Each type of electrode test was performed using three samples. The selected manufacturing technologies had been used in other electrochemical biosensor applications [17,18,19].

### 3.1. Cyclic Voltammetry Tests

**Figure 2:**
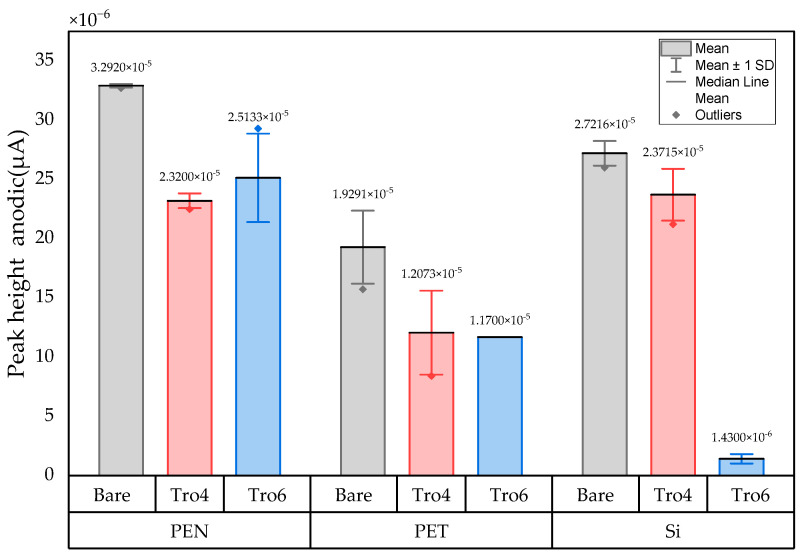
Mean and standard deviation of I_pa_ values of bare samples (gray), Tro4-functionalized samples (red) and Tro6-functionalized samples (blue) for PEN-, PET- and silicon-based substrates.

**Figure 3:**
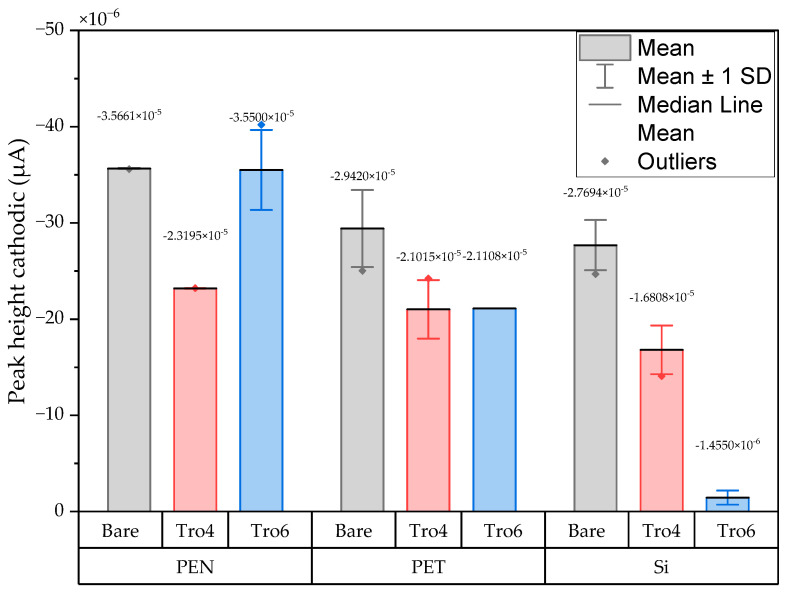
Mean and standard deviation of I_pc_ values of bare samples (gray), Tro4-functionalized samples (red) and Tro6-functionalized samples (blue) for PEN-, PET- and silicon-based substrates.

**Figure 4:**
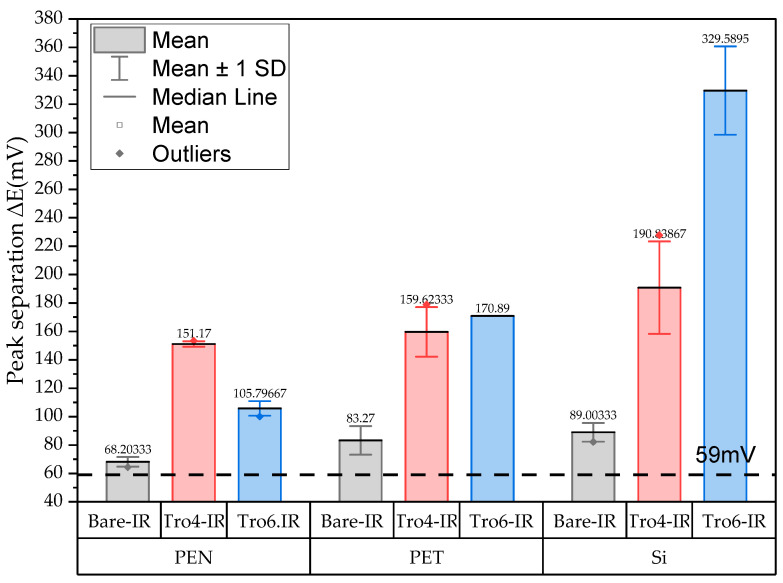
Mean and standard deviation of the peak separation (∆E) of bare samples (gray), Tro4-functionalized samples (red) and Tro6-functionalized samples (blue) for PEN-, PET- and silicon-based substrates.

The three conditions (bare samples (non-functionalized), Tro6-functionalized samples and Tro4Tro4-functionalized samples) were tested according to the parameters of the cyclic voltammetry (CV) experiments described in the Section 2.3. From the data generated by each test, the peak potential of both parts of the redox process as well as the peak heights of the redox process were collected as these are commonly used parameters from CV data [20,21]. The datum values of the anodic peak current (I_pa_) and cathodic peak current (I_pc_) for the PEN-based electrodes, silicon-based electrodes and PET-based electrodes of the samples are shown in Figure 2 and Figure 3, respectively. The I_pa_ and I_pc_ values obtained for each type of electrode were different due to the different morphologies, which were different from the measured geometric area [22]. All results were consistent with the literature for the solution-phase redox reporters hexacyanoferrade/hexacyaniferrade (K3 [Fe(CN)6]/K4[Fe (CN)6) and had the typical “duck” shape, where the peak shrinks due to the surface-modification blocking of the surface and the decrease in the current of the voltammogram [23,24]. The samples with the highest I_pa_ and I_pc_ for non-functionalized samples were the PEN-based samples. In second place were the silicon-based samples and those with a lower I_pa_ were the PET-based samples. The peak current ratios (I_pa_/I_pc_) were taken into account. For the reversible process used, it should ideally be equal to 1 for all samples at an equilibrium [25,26]. Taking into account the I_pa_ and I_pc_ values, we obtained I_pa_/I_pc_ values of 0.923171621, 0.655710401 and 0.982881907 for the PEN-based, PET-based and silicon-based samples, respectively. The PEN-based and silicon-based samples were closer to the ideal value, but the PET-based samples were far from it, which may have been due to the adhesion problems observed during the study. Based on the redox process considered in this study, the peak-to-peak separation (∆E) of the samples was analyzed against the theoretical value of 59.2/n mV (n = 1 at all scan rates, at 25 °C) [27]. Considering the values observed in Figure 4, the clean and non-functionalized PEN-based samples had a lower ∆E and were closer to the theoretical value. The sputtered gold layer on the silicon-based electrodes presented the highest peak separation ∆E of the sample types investigated. Based on this result, PEN-based samples might be more suitable for implementation in biosensors due to the highest basic output current signal and lower peak-to-peak separation, ensuring that the redox process of the reporter is not disturbed.

After obtaining the baselines for each type of sensor, they were functionalized using two cardiac troponin I-specific thiolated aptamers and 6-mercapto-1-heaxanol as a passivation agent, based on the functionalization protocol listed in the Section 2. CV techniques were performed for characterization. A summary of the results of the I_pa_, I_pc_ and ∆E are shown in Figure 2, Figure 3 and Figure 4, respectively. The CV plots used to obtain the I_pa_, I_pc_ and ∆E can be found in the Supplementary Materials. As reported in the literature, the aptamer and MCH acted as a blocking biolayer of the redox process, reducing the diffusion from the bulk solution to the surface [28,29,30]. The blocking of the transport process could be seen in the reduction in the I_pa_ and I_pc_, as shown in Figure 2 and Figure 3, and the increase in the ∆E was due to the drift in peak positions caused by the reduction in the electron-transfer process (see Figure 4) [31].

In general, the standard deviation was small for the whole set of cleaned samples. Once the samples had been functionalized using Tro4Tro4 and Tro6, the oxidation and reduction peaks decreased due to the blockade created by the aptamers, with their negatively charged backbone preventing the charge transfer from the bulk solution to the surface.

#### Cyclic Voltammetry of Modified PEN- and PET-Based Samples

Based on the results of the first batch of samples used, we made some modifications to the PET- and PEN-based samples. For the PEN-based samples, the change was to modify the surface area resulting from the standard manufacturing process that is used to give more uniformity and conductivity to a gold electrode [32,33,34,35]. In the case of the PET-based samples, the aim was to improve the adhesion of the gold layer to the PET substrate by adding an additional carbon or silver layer under the gold layer. Silver and carbon layers have been used on PET and the formation of gold on top has been possible for other applications such as transducers [13,36,37,38]. The surface morphology and adhesion current state of the gold surfaces for PEN and PET, respectively, are shown in Figure 4.

**Figure 5:**
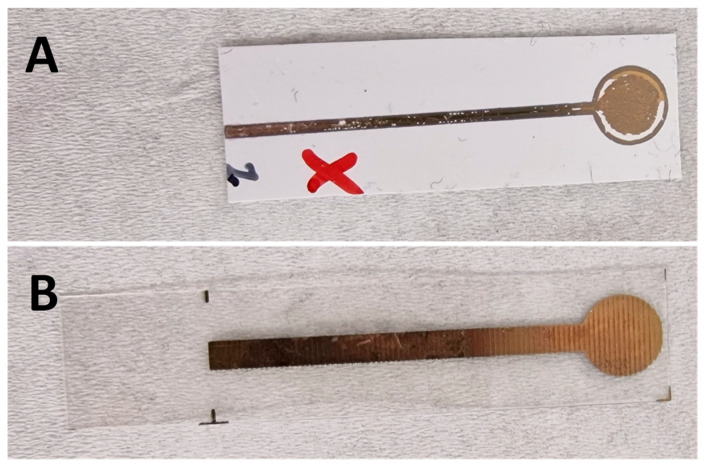
Examples of the initial batch of PET-based (**A**) and PEN-based (**B**) samples. Red “x” indicated samples that delaminated sections.

As shown in Figure 5A, the poor adhesion of the gold to the PET substrate was significant. In the sample shown, only the application of water released from the 1000 µL pipette used to rinse the samples was sufficient to remove part of the gold layer in some samples. Figure 5A,B show the lines on the gold-layer product of the inkjet-printing process, which affected the standard deviation values for the Tro4- and Tro6-functionalized samples.

**Figure 6:**
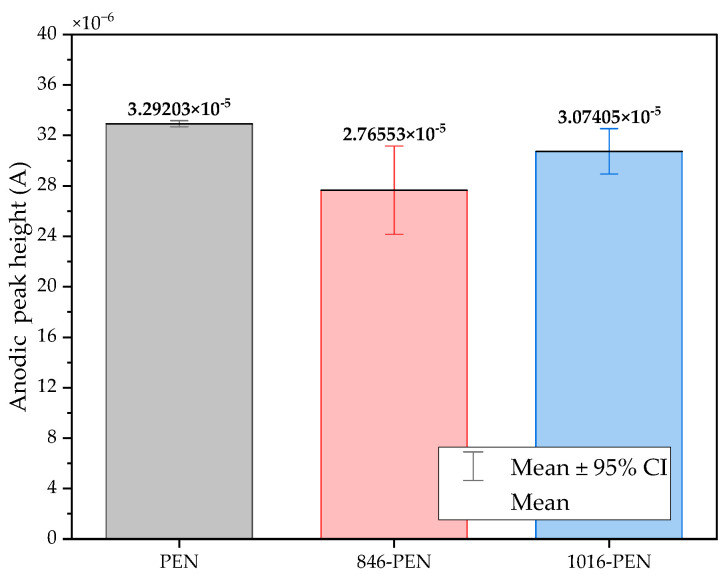
Mean and standard deviation of I_pa_ values of bare samples of unmodified PEN-based samples (gray), 846-PEN-based samples (red) and 1016-PEN-based samples (blue).

**Figure 7:**
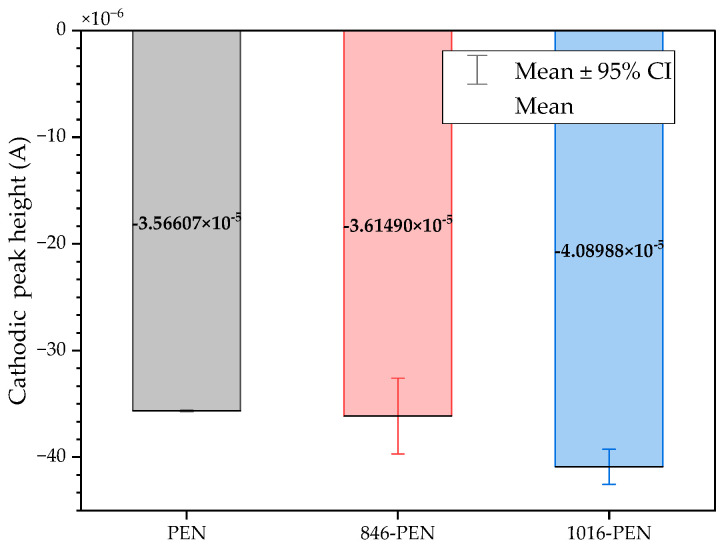
Mean and standard deviation of I_pc_ values of bare samples of unmodified PEN-based samples (gray), 846-PEN-based samples (red) and 1016-PEN-based samples (blue).

**Figure 8:**
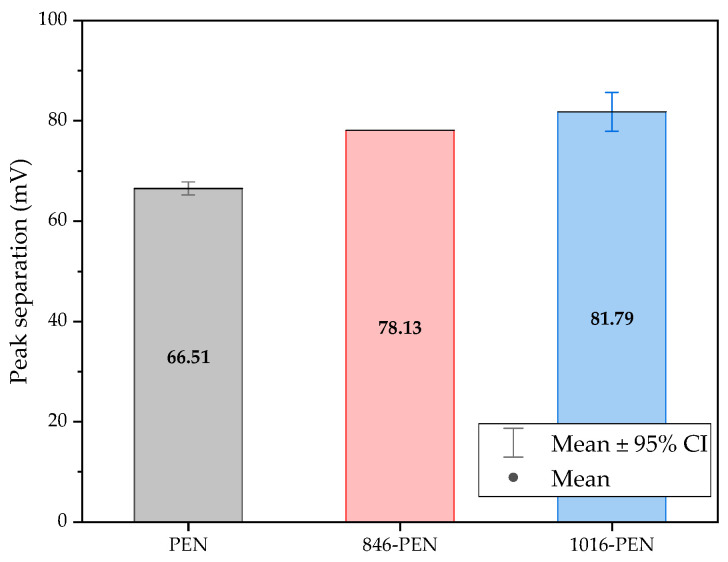
Mean and standard deviation of peak separation (∆E) values of bare samples of unmodified PEN-based samples, 846-PEN-based samples and 1016-PEN-based samples.

The morphology of the PEN-based samples was modified using a sintering process applied to two batches, one at 846 dpi and the other at 1016 dpi. To improve the adhesion of the gold to the PET-based samples, the new batches contained a silver layer under the gold layer for the first batch and a carbon layer for the second batch. All samples were electrochemically characterized. A summary of the CV results showing the I_pc_, I_pc_ and ∆E obtained from the new batches of sintered PEN, including the unsintered samples, are shown in Figure 6, Figure 7 and Figure 8 respectively.

The results showed that the I_pa_ decreased and the I_pc_ increased for both the 846 dpi and 1016 dpi samples compared with the non-sintered samples. The SD also increased. In the case of the peak current ratio (I_pa_/I_pc_), the values were 0.921, 0.765 and 0.758 for the non-sintered, 846 and 1016 samples, respectively, which were not desirable results. Based on these results, modifications using a sintering process affected the reversibility of the redox process, based on the I_pa_/I_pc_ values of the samples. Considering the peak separation, we observed that the reversibility of the redox process used for characterization was better for the non-sintered samples as we observed a lower ∆E.

**Figure 9:**
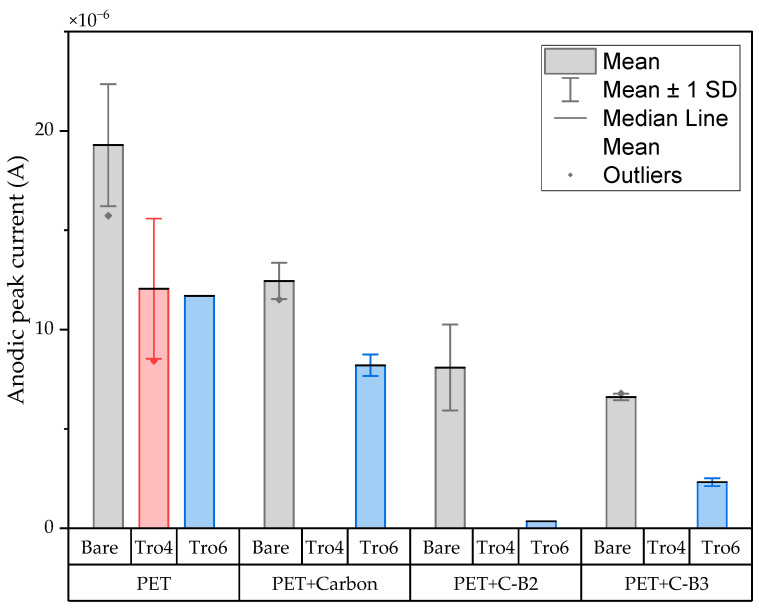
Mean and standard deviation of I_pa_ values of bare samples (gray), Tro4-functionalized samples (red) and Tro6-functionalized samples (blue) for carbon-modified PET-based samples from the initial batch and 3 other batches.

**Figure 10:**
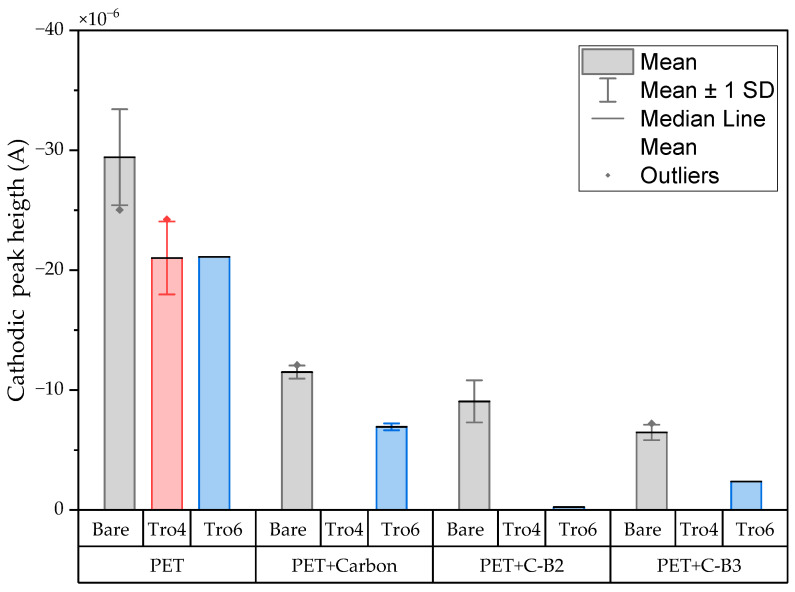
Mean and standard deviation of I_pc_ values of bare samples (gray), Tro4-functionalized samples (red) and Tro6-functionalized samples (blue) for carbon-modified PET-based samples from the initial batch and 3 other batches.

**Figure 11:**
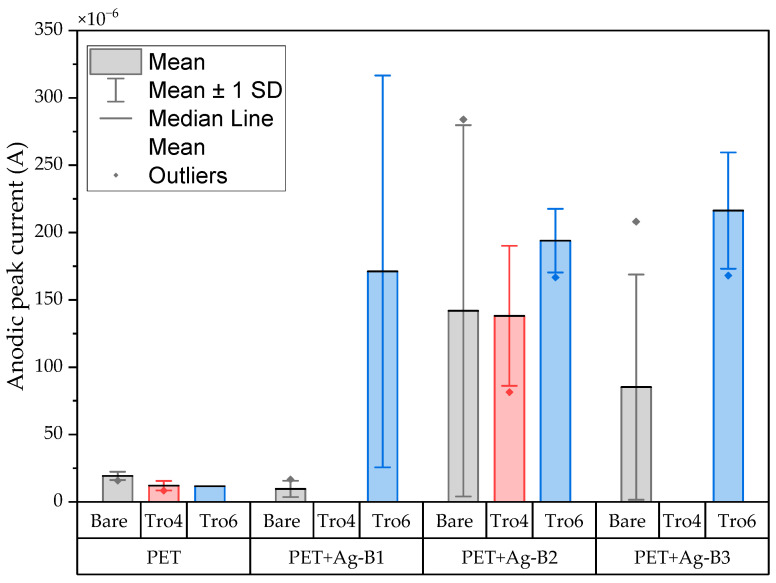
Mean and standard deviation of I_pa_ values of bare samples (gray), Tro4-functionalized samples (red) and Tro6-functionalized samples (blue) for silver-modified PET-based samples from the initial batch and 3 other batches.

The CV results of the modified samples with carbon are shown in Figure 9 and Figure 10 and the CV results of the modified samples with silver are shown in Figure 11. Based on the data in Figure 8 and Figure 10, the I_pa_ and I_pc_ values for the non-functionalized and functionalized samples were higher for the unmodified samples than for the samples with a carbon layer. For the Tro4Tro4-functionalized samples with a carbon layer, a peak search to determine the I_pa_ and I_pc_ was not achievable for the software, hence the columns are not visible in the figures.

**Figure 12:**
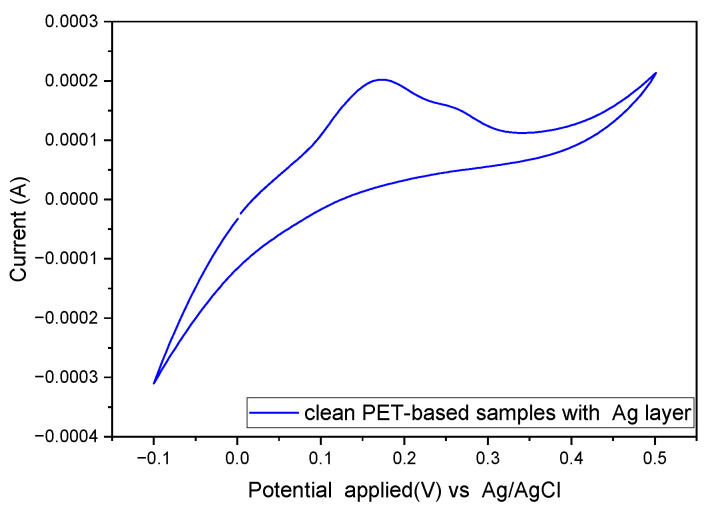
Cyclic voltammogram of PET-based sample with a silver layer under a gold layer in a standard PBS buffer containing 1 mM [Fe (CN)6]^3−/4−^.

The PET-based samples modified with a silver layer presented greater limitations in the obtention of CV parameters due to the recorded current values from the CV experiment. The I_pa_ and SD values of the clean samples in batch two increased and this trend was also observed in the functionalized samples for the three batches with a silver layer. It was not possible to estimate the I_pc_ and ∆E from the available datasets of the samples tested with a silver layer. An example cyclic voltammogram for these samples is shown in Figure 12.

### 3.2. Electrochemical Impedance Spectroscopy (EIS)

**Figure 13:**
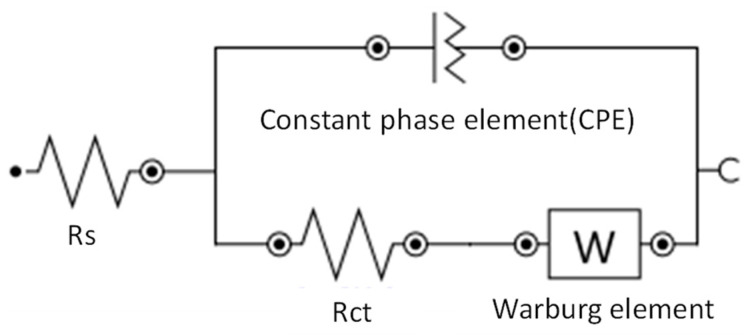
Modified Randle’s equivalent circuit.

**Figure 14:**
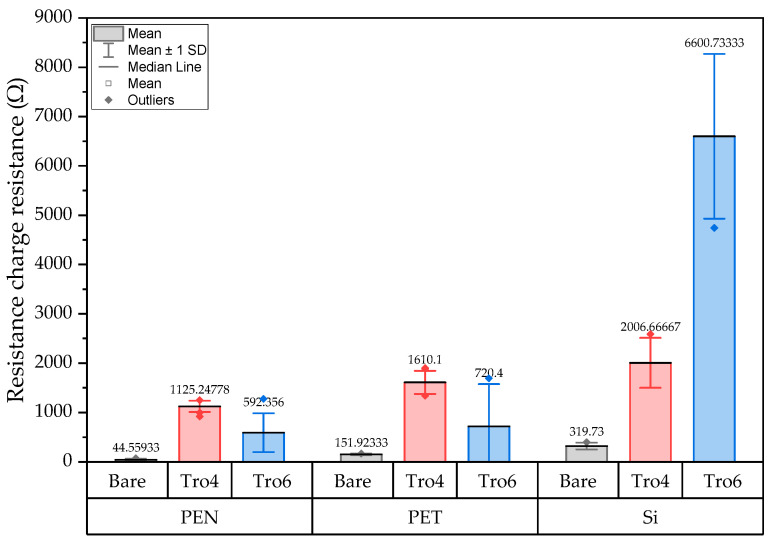
Mean and standard deviation of the Rct values of bare samples (gray), Tro4-functionalized samples (red) and Tro6-functionalized samples (blue) for PEN-, PET- and silicon-based substrates.

EIS was carried out to complement and corroborate the CV results. The tests were carried out using the same samples as in the CV section and the parameter we focused on was the charge-transfer resistance, which was obtained by fitting the impedance spectra generated in the EIS experiment to a modified Randle’s equivalent circuit and then presenting the results in a Nyquist plot. An example of a modified Randle’s equivalent circuit is shown in Figure 13 [27], where Rs is the resistance of the solution, Rct is the charge-transfer resistance, CPE is the constant phase element and W is the Warburg impedance. The fitting of the impedance spectra to such a model was carried out using NOVA software (version 2.1.5) from Metrohm Autolab. The fitted charge-transfer resistance (Rct) value was used for the analysis as it is commonly used in the literature [39]. The first set of samples comprised unmodified PEN-based, PET-based and Si-based samples and a summary of the Rct values of the different samples is shown in Figure 14. The Nyquist plots used to obtain the Rct values can be found in the Supplementary Materials.

**Figure 15:**
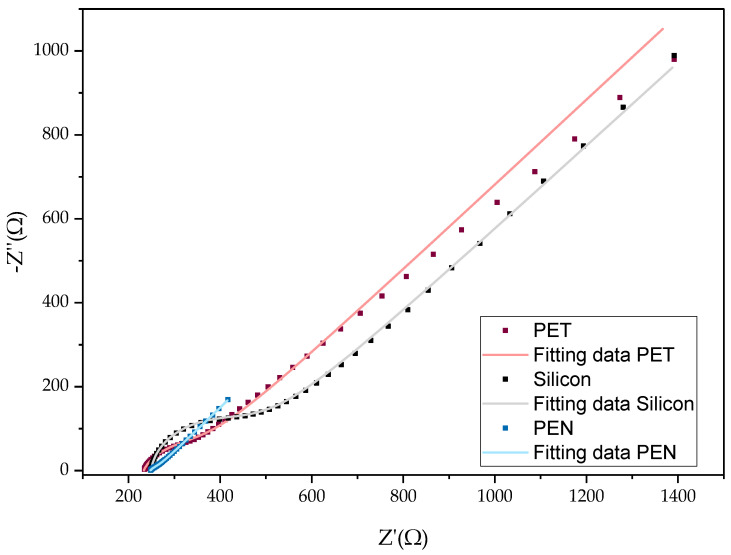
Nyquist plots of 3 individual samples after a cleaning step recorded in standard PBS buffer containing 1 mM [Fe (CN)6]^3−/4−^ at OCP. Blue dotted line: experimental data of inkjet-printed gold layer on PEN and blue line fitted data. Red dotted line: experimental data of screen-printed gold layer on PET and red line fitted data. Black dotted line: experimental data of sputtered gold layer on silicon oxide wafer and black line fitted data.

Based on the EIS results shown in Figure 14, the Rct value of the PEN-based samples showed a lower Rct for the bare samples. The bare samples with the highest Rct values were the silicon-based samples, with an average value of 319.73 Ω. For the PET-based samples, the Rct value was three times higher than the PEN samples. When fitting the impedance spectra for the PEN-based sample, the semicircle resulting from the parallel arrangement of the Rct and CPE values in the circuit could not be well-observed, as shown in Figure 15 (blue line and blue dotted line). The NOVA software required more time and better starting values of the electrical circuit in the simulation to obtain a better fit of the data [40]. In comparison, the Rct values of the PET-based and silicon-based samples (Figure 15 red curves and black curves respectively) were obtained faster and with a smaller modification of the initial values used. The impedance spectra of the bare PEN-based samples showed a Faradic process, mainly driven by the diffusion of ions to the surface.

The Tro4- and Tro6-functionalized surfaces showed an increased charge-transfer resistance for all samples, as shown in Figure 14. The values of the Tro4-functionalized sample showed a lower standard deviation (SD) compared with the values of the Tro6-functionalized sample.

We used the percentage of change of the charge-transfer resistance (∆Rct (%)) to better understand the changes. ∆Rct (%) could be calculated using Equation (1), as follows:

(1) $$∆Rct\;\left(\%\right)=\frac{R_{ct}final-R_{ct}initial}{R_{ct}initial}\times 100\%$$

where $R_{ct}final$ represents the Rct value after functionalization and $R_{ct}initial,$ represents the Rct value of the bare samples. The obtained ∆Rct (%) figures are presented in Table 1. The Tro4-functionalized samples had the greatest change for the PEN-based samples, followed by the PET-based samples and then, with the lowest change, the silicon-based samples, at 528% ∆Rct (%). Tro6 functionalization with the highest ∆Rct (%) was obtained with the silicon-based samples, followed by the PEN-based samples; the lowest ∆Rct (%) values were from the PET-based samples. The silicon-based samples had a higher ∆Rct (%) with Tro6 functionalization; however, the SD was high. The PET-based samples showed a high SD for both functionalizations.

#### Electrochemical Impedance Spectroscopy of Modified PEN- and PET-Based Samples

**Figure 16:**
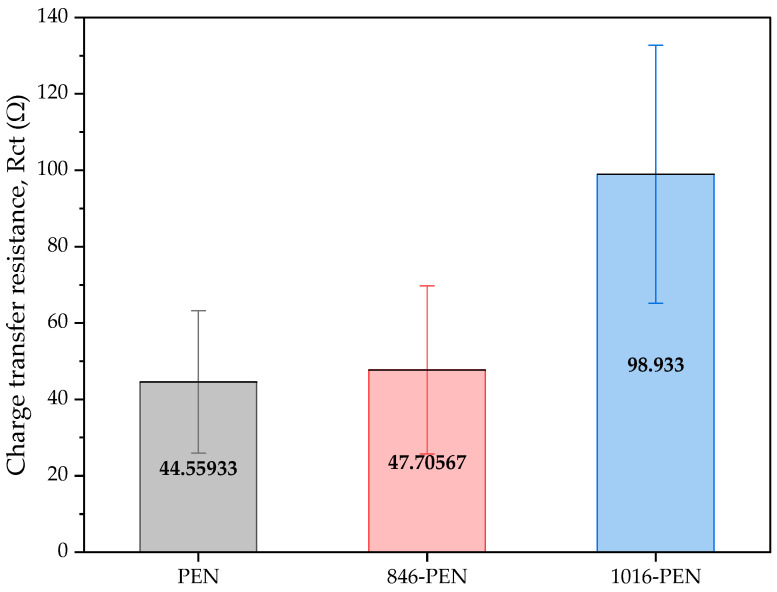
Mean and standard deviation of the Rct values of bare samples of unmodified PEN-based samples, 846-PEN-based samples and 1016-PEN-based samples.

The EIS characterization parameters for the modified PEN-based and PET-based samples were the same as for the first batch. The results for the modified PEN-based samples without functionalization are shown in Figure 16. The results showed that the initial Rct increased for the sintered samples, but the semicircle where the kinetic control had an influence remained the same as the example shown in Figure 15.

**Figure 17:**
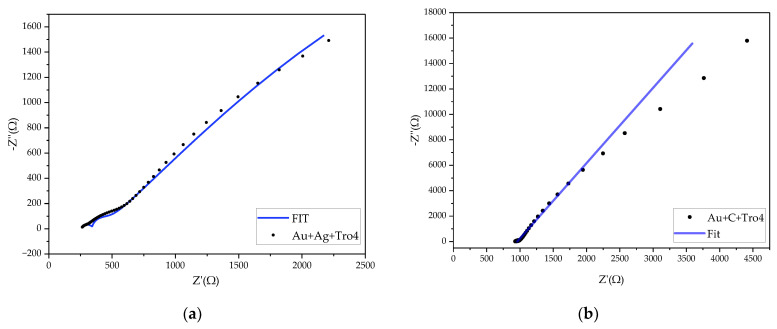
Nyquist plots of modified PET-based samples with Tro4 functionalization. (**a**) Nyquist plot of PET samples with an Ag layer under the gold and functionalized using a Tro4 aptamer and (**b**) Nyquist plot of PET samples with a C layer under the gold and functionalized using a Tro4 aptamer.

The impedance spectra of the modified PET samples with either a carbon or a silver layer showed several changes compared with the unmodified samples, which interfered with the fitting of the data to the initial electrical model used previously. Two impedance spectrum datum examples from the characterized samples are shown in Figure 17. The impedance data suggested that the electrochemical system had a different layer configuration compared with the proposed Randle’s equivalent circuit. The data showed two semicircles, which altered the electrical model required for fitting [40]. The modelling of a new electrical circuit, which deviated from the proposed idea that the new layer only improved the adhesion of the gold layer to the substrate without affecting the EC parameters, was not possible with the manufacturing process used. The Supplementary Materials presents the Nyquist plots of the experimental data for the modified PET-based samples with silver and carbon layers.

## 4. Discussion

The electrochemical characterization of the three types of samples before and after functionalization was achievable and the overall results for the three types of samples had comparable outputs in the CV and EIS experiments, where the inclusion of an aptamer–MCH layer affected the electron transfer of the surface due to the negatively charged aptamer [41].

The respective change in Rct for each sample type varied based on the aptamer, the aptamer concentration and the working electrode used. In the literature, the functionalization of Tro4 and Tro6 thiolated aptamers on electrodes was demonstrated to be possible on three types of samples at different levels due to the surface morphology [42,43]. The change in Rct in the literature was the result of reducing the electron-transfer process, which increased the Rct values, reduced the I_pc_–I_pa_ values and increased the ∆E [44,45,46]. As described in the literature, each manufacturing technology has different baseline values depending on the functionalization protocol and the specific aptamer used, as observed in this study where the same functionalization protocol was used for two cardiac troponin I aptamers. The bare samples showed a peak-to-peak separation close to the theoretical value for a reversible process and these values were similar to those found in the literature for each type of manufacturing technology [44,47,48]. From the three types of samples, the peak separation of the PEN-based samples was the lower (68 mV) by at least 10 mV with respect to the other two types of samples, indicating better conditions of the reversibility of the process. Having lower starting ∆E values helps because the incorporation of aptamers is known to slow the electron transfer even further. The smaller shift of the current peaks could have a lower impact on the aptamer–MCH layer for the long-term use of electrodes with voltametric approaches by applying smaller potential windows with lower potentials. In a similar manner, having higher baseline I_pc_ and I_pa_ values, as was the case for the PEN samples, could help improve the resolution in the further developmental steps of electrochemical biosensors [49]. Once the aptamers were immobilized, the CV analysis indicated that the PEN-based samples had low variations between samples and the current values were higher for both functionalizations using PEN-based samples. Such baselines are preferable for the further development of sensors in POCT applications or wearable applications where the robustness of the readout system is lower than that of a laboratory setup. With respect to the EIS measurements, many examples have shown ∆Rct (%) values from 200% to 600% for aptamer concentrations ranging from 0.5 µM to 15 µM [19,50], which are indeed smaller than the values of ∆Rct (%) obtained for the 25 µM aptamer concentration used in this study, based on an increase in aptamer density at the surface. Other baseline Rct values that are different from those investigated here can be found in the literature for different gold electrodes [9,40,41,43]. Similar to the CV analysis, the EIS showed that the PEN-based samples had higher Rct changes while maintaining a lower sample-to-sample variation for the Tro4-functionalized samples and Tro6-functionalized samples, a quality that will be helpful with further surface modifications and detection experiments where possible target interactions cannot be distinguished because the standard deviation of the values used as a baseline overshadows these small changes.

For the first set of samples tested, the EIS response was smooth and had easy parametrization when compared with other working electrodes in the literature [51]. However, once the modified versions of the PEN and PET samples were studied, the sintering process for the PEN-based samples did not reduce the Rct values, as in most cases in the literature [52]. In the case of the PET-based samples, a different equivalent circuit was required due to the new active layer configuration that the system produced during the characterization. This may have occurred due to the aptamer and MCH layer, as reported in other literature, although this could not be the case as the other samples tested in this study did not have such results [53].

The reproducibility of the modified PET-based samples after functionalization suggested that the uncontrolled roughness hindered the adhesion of the thiol anchor, which affected the uniformity and density of the aptamer + MCH layer. This was noticeable in the increase in SD and low ∆Rct (%) when the functionalized samples were characterized. It is also important to mention that the addition of a carbon or silver layer produced unexpected results when compared with examples in the literature where glassy carbon electrodes were presented with a gold layer on top, which showed good conductivity, and no modification models were used for the EIS analysis, something that was not the case in this study. In the case of the silver-modified electrodes, the electrochemical test results could be attributed to the oxidation of the silver layer. Such a layer should not be in contact with the electrochemical solution to avoid this interaction [54].

The improvement in the metal–polymer adhesion could be tackled using other strategies such as using an argon treatment instead of adding an extra layer [55,56]. With the current results, the use of carbon- or silver-modified samples was not a comparable option with other cases in the literature. In the case of the silicon-based option, the example that we found in the literature could be compared in the non-functionalized state; however, the introduction of the aptamer had a lower priority compared with the PEN-based option [47]. A low variability between samples for each electrode technology relative to the baseline measurements taken is something that has been obtained in other studies for biological fluids and at a more fundamental level [57]. The results presented in this study suggested that further testing and optimization are required to ensure a low variability so that transducers are suitable for aptamer sensor applications; however, the results showed a good starting point for the electrodes tested.

## 5. Conclusions

Based on an electrochemical characterization, PEN-based samples were the easiest to characterize compared with PET- and silicon-based samples due to the artefact presented in the electrochemical data where the redox process was not fully observed in the initial potential window used. The PEN-based samples also showed higher current values in the CV and lower peak separation. In the case of the PET samples, the variation in the EC parameter of the measured samples and the consistency of the prepared samples were not suitable for a better EC characterization, mainly due to the delamination of the gold layer, which changed the ECSA.

Once the aptamers Tro4 and Tro6 were introduced to the surface by immobilization with a thiol anchor, the PEN-based samples showed a greater change in ∆Rct (%) values with respect to the non-functionalized samples for Tro4. In second place was the Tro6 aptamer, indicating that there was greater immobilization on the surface of these samples, which correlated with a greater number of aptamers on the surface blocking the electron-transfer process. The modifications presented in this study for the PEN- and PET-based samples did not improve the results with the immobilization of aptamers as we had proposed because the electrochemical characterization showed that the different EC parameter values obtained were deficient when compared with the unmodified samples for most of the parameters used in this study, which made them less suitable for aptamer immobilization and the detection of cardiac troponin I using a label-free approach compared with the unmodified samples.

Although the PEN-based samples gave better results, this technology should be improved to reduce the variation between samples and to propose surface modifications to improve the electrochemical parameters. Possible modifications include the addition of nanomaterials such as carbon nanotubes or other nanoparticles such as hematite to the surface, which will improve the electrochemical properties of the surface. Another modification that can be made to the substrate is a specific morphology and roughness so that the gold layer on top changes accordingly and the ECSA could be modified in a positive way. Other sintering protocols can also be considered to improve conductivity and reduce the sample-to-sample variation. These modifications should maintain the suitability of the surface to immobilize Tro4 and Tro6 aptamers for their use in detection experiments for cardiac troponin I.

## Figures and Tables

**Table 1:** ΔRct (%) values for the different groups of samples tested after immobilization with the aptamers Tro4 and Tro6.

∆Rct (%)	PEN	PET	Silicon
**Tro4**	2425.28%	960%	528%
**Tro6**	1229%	374%	1964%

## Data Availability

The original contributions presented in the study are included in the article/Supplementary Materials; further inquiries can be directed to the corresponding author.

## Supplementary Materials

The following supporting information can be downloaded at: https://www.mdpi.com/article/10.3390/bios14070341/s1.
