# Supplementary material for: Evaluation of Transducer Elements Based on Different Material Configurations for Aptamer-Based Electrochemical Biosensors
Source: Biosensors (Basel). 2024 Jul 13;14(7):341. doi: 10.3390/bios14070341 (PMC11274616; doi:10.3390/bios14070341)
Supplement: Supplementary file 1 [file biosensors-14-00341-s001.zip › biosensors-3065918-supplementary.pdf]

biosensors

MDPI

Supplementary Materials

Evaluation of Transducer Elements Based on Different Material Configurations for Aptamer-Based Electrochemical Biosensors

Ivan Lopez Carrasco [1], Gianaurelio Cuniberti [2], Jörg Opitz [1] and Natalia Beshchasna [1,*]

[1] Fraunhofer Institute for Ceramic Technologies and Systems IKTS, Maria-Reiche-Strasse 2, 01109 Dresden, Germany; ivan.lopez.carrasco@ikts.fraunhofer.com (I.L.C.); joerg.opitz@ikts.fraunhofer.de (J.O.)

2 Faculty of Mechanical Science and Engineering, Institute of Materials Science and Max Bergmann Center of Biomaterials, Technische Universität Dresden, 01062 Dresden, Germany; office.nano@tu-dresden.de

* Correspondence: natalia.beshchasna@ikts.fraunhofer.de

Content

Figure S1. Experimental CV data of bare samples, PEN-based samples (black), PET-based samples(red) and silicon-based samples (blue). Recorded in PBS solution with 1mM [Fe (CN)6] $^{3-/4}$. Continues line sample 1, dash line samples 2 and dot line sample 3 for each type of electrodes. 2

Figure S2. Experimental CV data of tro4-functionalized samples, PEN-based samples (black), PET-based samples(red) and silicon-based samples (blue). Recorded in PBS solution with 1mM [Fe (CN)6] $^{3-/4}$. Continues line sample 1, dash line samples 2 and dot line sample 3 for each type of electrodes. 3

Figure S3. Experimental CV data of tro6-functionalized samples, PEN-based samples (black) PET-based samples(red) and silicon-based samples (blue). Recorded in PBS solution with 1mM [Fe (CN)6] $^{3-/4}$. Continues line sample 1, dash line samples 2 and dot line sample 3 for each type of electrodes. 3

Figure S4. Experimental CV data of bare samples, PEN-based samples (black), 846-PEN-based samples(red) and 1016-PEN-based samples (blue). Recorded in PBS solution with 1mM [Fe (CN)6] $^{3-/4}$. Continues line sample 1, dash line samples 2 and dot line sample 3 for each type of electrodes. 4

Figure S5. Experimental CV data of carbon modified PET-based samples. Each curve is the CV data from one sample; continues line sample 1, dash line sample 2 and dotted line sample 3. The different colours indicate different sets of samples. Recorded in PBS solution with 1mM [Fe (CN)6] $^{3-/4}$. 4

Figure S6. Experimental CV data of silver modified PET-based samples. Each curve is the CV data from one sample; continues line sample 1, dash line sample 2 and dotted line sample 3. The different colours indicate different sets of samples. Recorded in PBS solution with 1mM [Fe (CN)6] $^{3-/4}$. 5

Figure S7. Experimental EIS data of bare samples, PEN-based samples (black) PET-based samples(red) and silicon-based samples (blue). Recorded in PBS solution with 1mM [Fe (CN)6] $^{3-/4}$. Continues line sample 1, dash line samples 2 and dot line sample 3 for each type of electrodes. 5

Figure S8. Experimental EIS data of tro4-functionalized samples, PEN-based samples (black) PET-based samples(red) and silicon-based samples (blue). Recorded in PBS

solution with 1mM [Fe (CN)6] $^{3-/4}$. Continues line sample 1, dash line samples 2 and dot line sample 3 for each type of electrodes. 6

Figure S9. Experimental EIS data of tro6-functionalized samples, PEN-based samples (black) PET-based samples(red) and silicon-based samples (blue). Recorded in PBS solution with 1mM [Fe (CN)6] $^{3-/4}$. Continues line sample 1, dash line samples 2 and dot line sample 3 for each type of electrodes. 6

Figure S10. Nyquist plots of experimental EIS data of carbon modified PET-based samples. Each curve is the CV data from one sample; continues line sample 1, dash line sample 2 and dotted line sample 3. The different colours indicate different sets of samples. Recorded in PBS solution with 1mM [Fe (CN)6] $^{3-/4}$. 7

Figure S11. Nyquist plots of experimental EIS data of silver modified PET-based samples. Each curve is the CV data from one sample; continues line sample 1, dash line sample 2 and dotted line sample 3. The different colours indicate different sets of samples. Recorded in PBS solution with 1mM [Fe (CN)6] $^{3-/4}$. 7

Figure S12. Experimental EIS data of bare samples, PEN-based samples (black), 846-PEN-based samples(red) and 1016-PEN-based samples (blue). Recorded in PBS solution with 1mM [Fe (CN)6] $^{3-/4}$. Continues line sample 1, dash line samples 2 and dot line sample 3 for each type of electrodes. 8

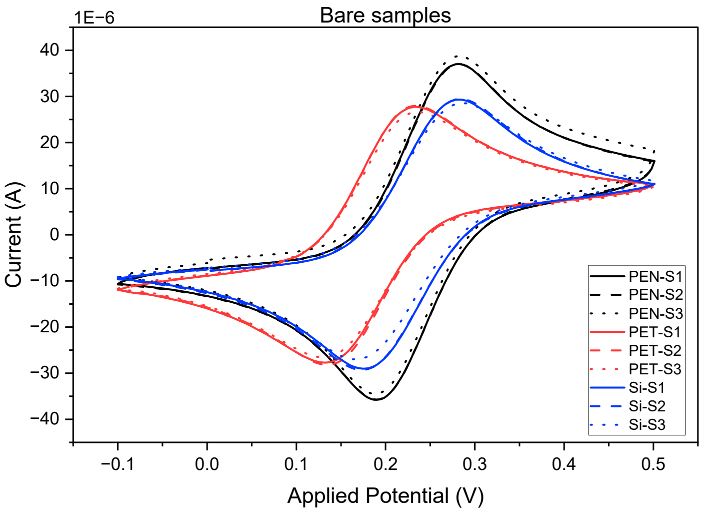

Figure S1. Experimental CV data of bare samples, PEN-based samples (black), PET-based samples(red) and silicon-based samples (blue). Recorded in PBS solution with 1mM [Fe (CN)6] $^{3-/4}$. Continues line sample 1, dash line samples 2 and dot line sample 3 for each type of electrodes.

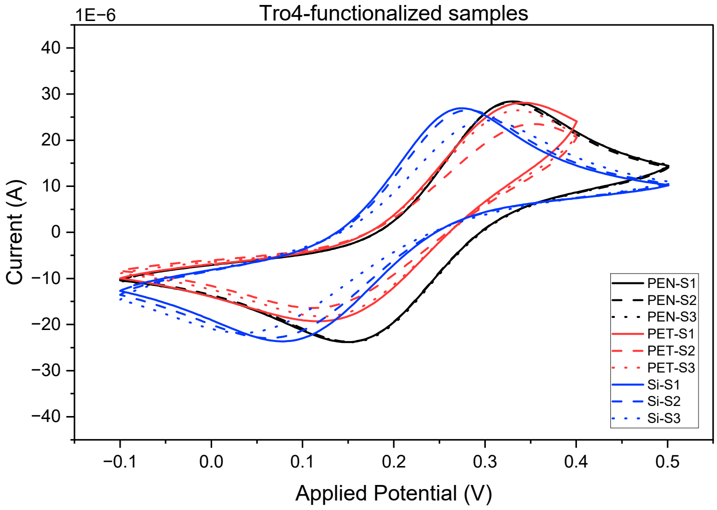

Figure S2. Experimental CV data of tro4-functionalized samples, PEN-based samples (black), PET-based samples(red) and silicon-based samples (blue). Recorded in PBS solution with 1mM [Fe (CN)6] $^{3-/4}$. Continues line sample 1, dash line samples 2 and dot line sample 3 for each type of electrodes.

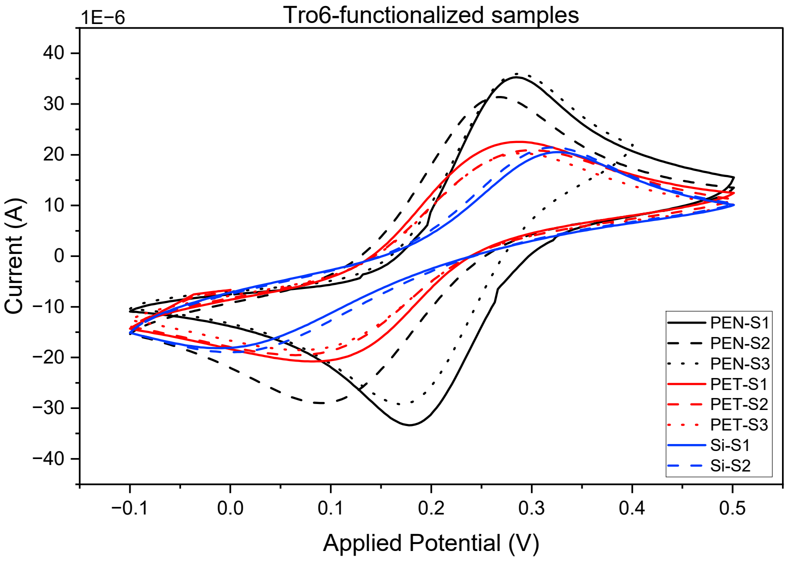

Figure S3. Experimental CV data of tro6-functionalized samples, PEN-based samples (black) PET-based samples(red) and silicon-based samples (blue). Recorded in PBS solution with 1mM [Fe (CN)6] $^{3-/4}$. Continues line sample 1, dash line samples 2 and dot line sample 3 for each type of electrodes.

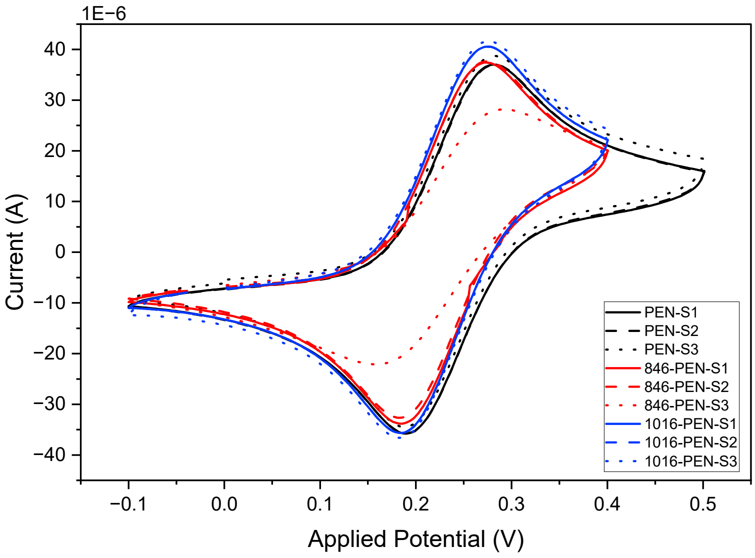

Figure S4. Experimental CV data of bare samples, PEN-based samples (black), 846-PEN-based samples(red) and 1016-PEN-based samples (blue). Recorded in PBS solution with 1mM [Fe (CN)6] $^{3-/4}$. Continues line sample 1, dash line samples 2 and dot line sample 3 for each type of electrodes.

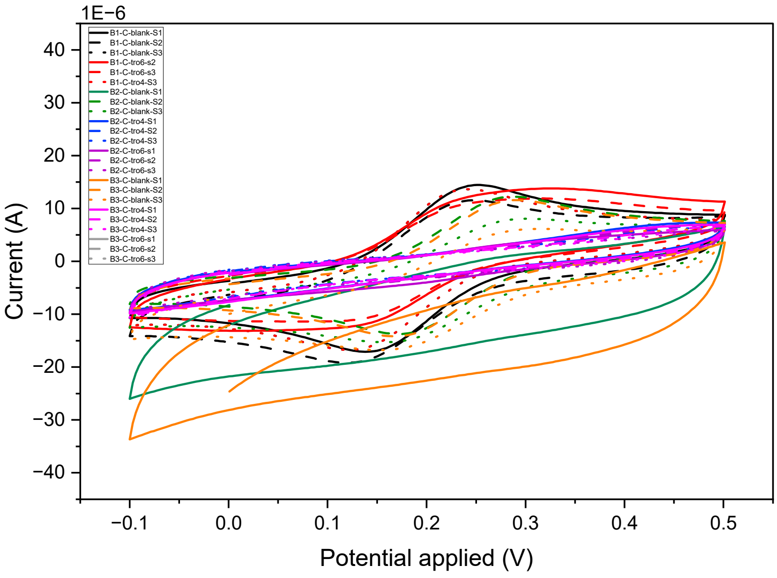

Figure S5. Experimental CV data of carbon modified PET-based samples. Each curve is the CV data from one sample; continues line sample 1, dash line sample 2 and dotted line sample 3. The different colours indicate different sets of samples. Recorded in PBS solution with 1mM [Fe (CN)6] $^{3-/4}$.

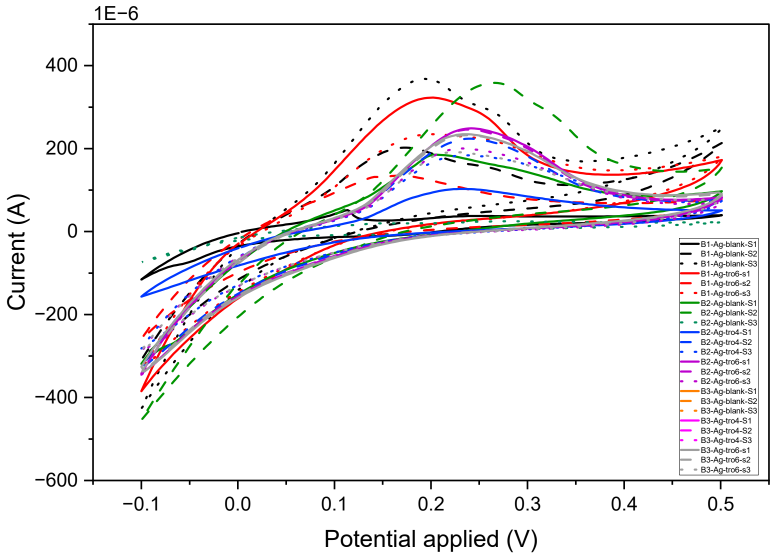

Figure S6. Experimental CV data of silver modified PET-based samples. Each curve is the CV data from one sample; continues line sample 1, dash line sample 2 and dotted line sample 3. The different colours indicate different sets of samples. Recorded in PBS solution with 1mM [Fe (CN)6] $^{3-/4}$.

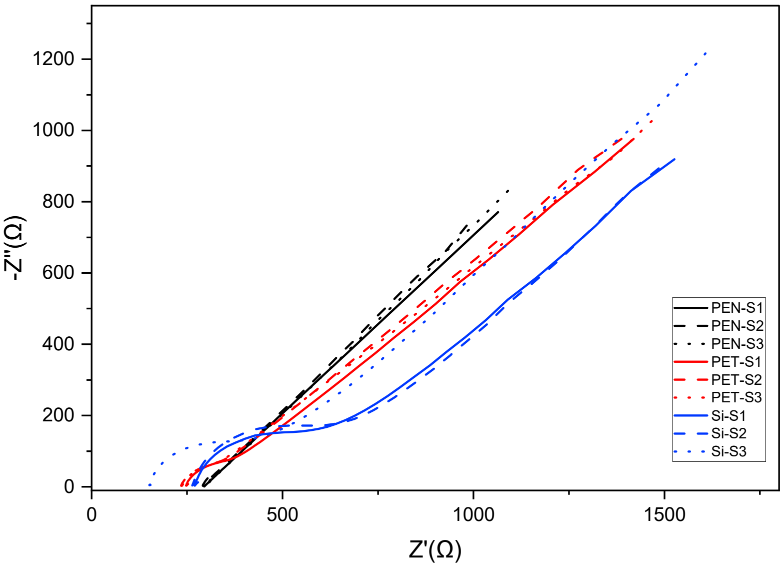

Figure S7. Experimental EIS data of bare samples, PEN-based samples (black) PET-based samples(red) and silicon-based samples (blue). Recorded in PBS solution with 1mM [Fe (CN)6] $^{3-/4}$. Continues line sample 1, dash line samples 2 and dot line sample 3 for each type of electrodes.

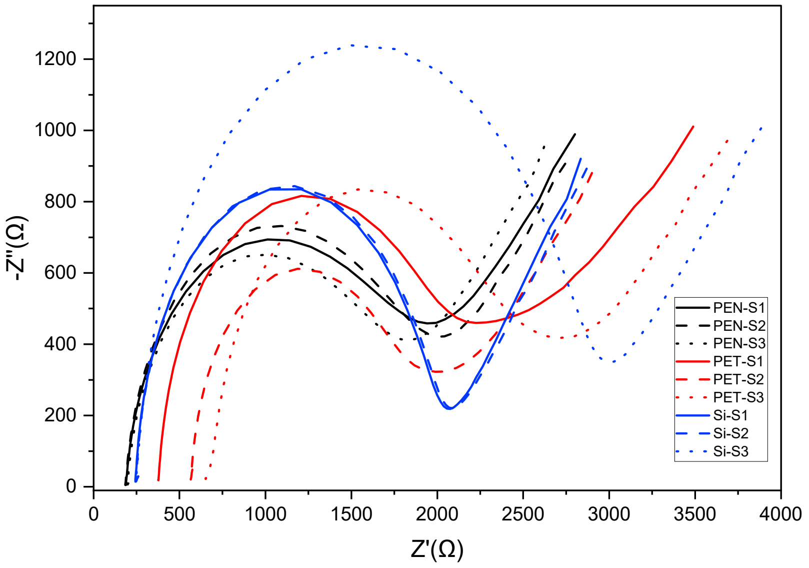

Figure S8. Experimental EIS data of tro4-functionalized samples, PEN-based samples (black) PET-based samples(red) and silicon-based samples (blue). Recorded in PBS solution with 1mM [Fe (CN)6] $^{3-/4}$. Continues line sample 1, dash line samples 2 and dot line sample 3 for each type of electrodes.

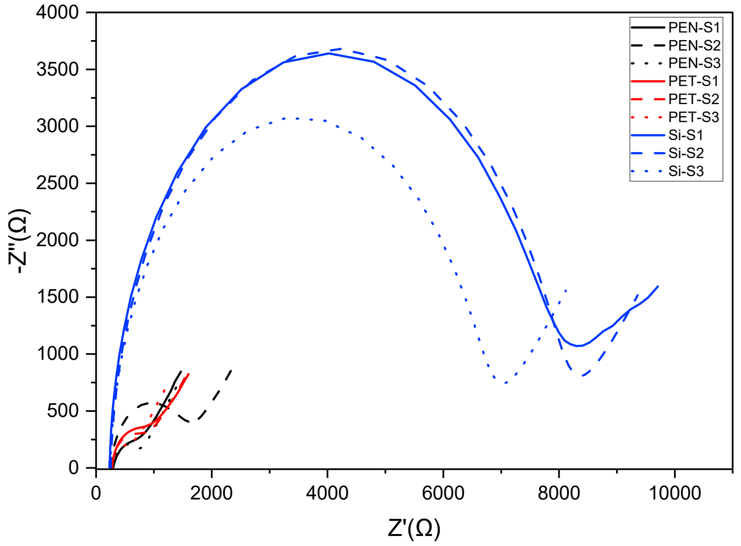

Figure S9. Experimental EIS data of tro6-functionalized samples, PEN-based samples (black) PET-based samples(red) and silicon-based samples (blue). Recorded in PBS solution with 1mM [Fe (CN)6] $^{3-/4}$. Continues line sample 1, dash line samples 2 and dot line sample 3 for each type of electrodes.

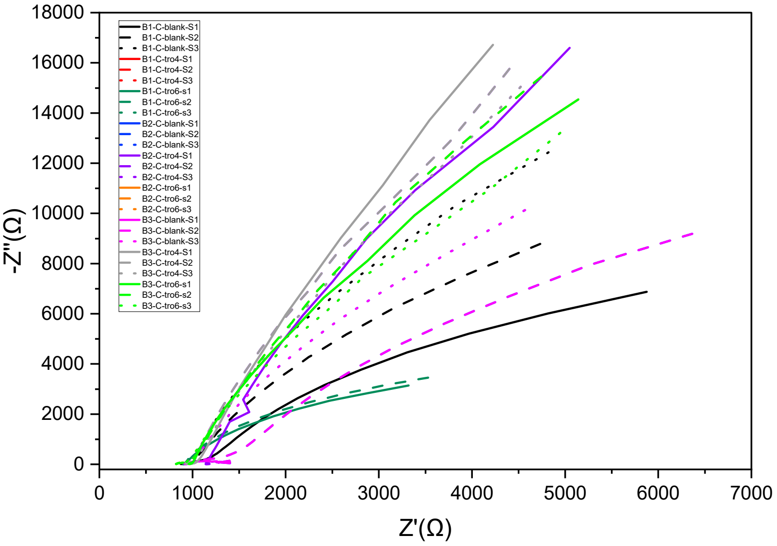

Figure S10. Nyquist plots of experimental EIS data of carbon modified PET-based samples. Each curve is the CV data from one sample; continues line sample 1, dash line sample 2 and dotted line sample 3. The different colours indicate different sets of samples. Recorded in PBS solution with 1mM [Fe (CN)6] $^{3-/4}$.

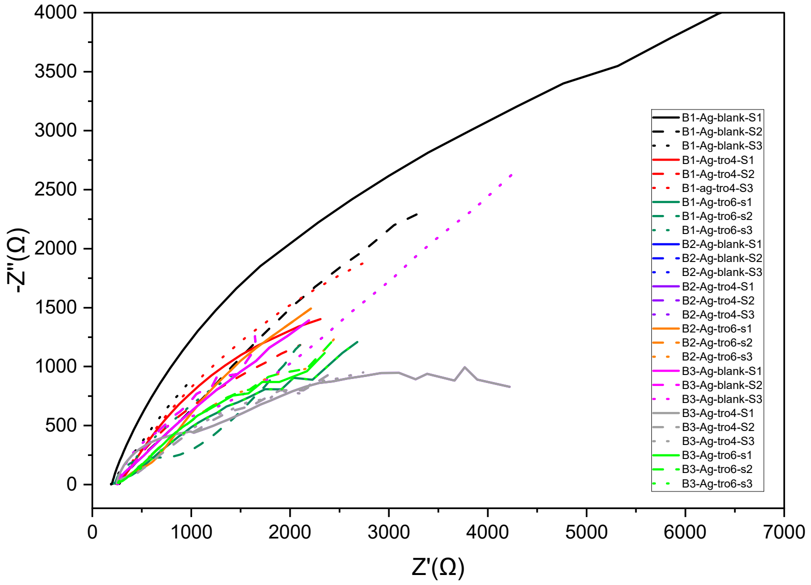

Figure S11. Nyquist plots of experimental EIS data of silver modified PET-based samples. Each curve is the CV data from one sample; continues line sample 1, dash line sample 2 and dotted line sample 3. The different colours indicate different sets of samples. Recorded in PBS solution with 1mM [Fe (CN)6] $^{3-/4}$.

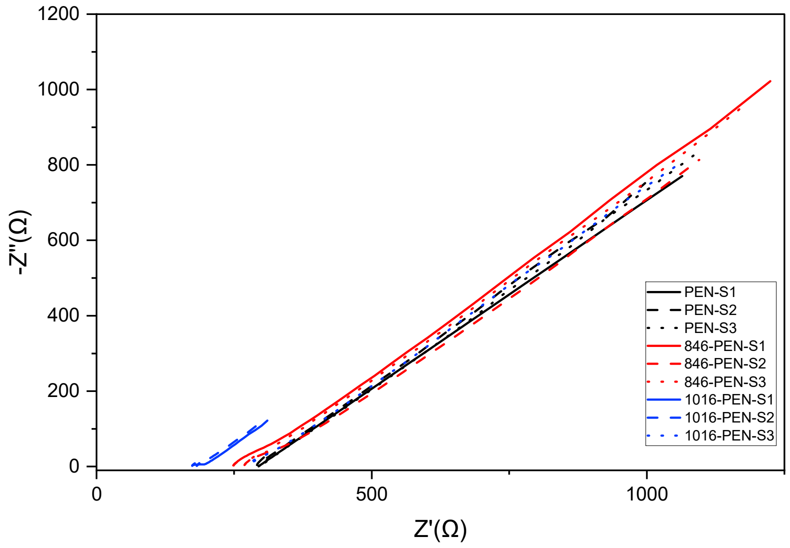

Figure S12. Experimental EIS data of bare samples, PEN-based samples (black), 846-PEN-based samples(red) and 1016-PEN-based samples (blue). Recorded in PBS solution with 1mM [Fe (CN)6] $^{3-/4}$. Continues line sample 1, dash line samples 2 and dot line sample 3 for each type of electrodes.
